# Supplementary material for: Human glycogenins maintain glucose homeostasis by regulating glycogen metabolism
Source: Nat Commun. 2025 Jul 16;16:6556. doi: 10.1038/s41467-025-61862-3 (PMC12267440; doi:10.1038/s41467-025-61862-3)
Supplement: Supplementary file 1 — Supplementary Information [file 41467_2025_61862_MOESM1_ESM.pdf]

Supplementary Information

Human glycogenins maintain glucose homeostasis by regulating glycogen metabolism

Authors: Tzu-Han Weng[1], Yu-Chung Pien[1,#], Ching-Jou Chen[1,#], Po-Pang Chen[2, #], Yu-Ting Tseng[2], Ying-Chen Chen[1], Wen-Po Hsiao[1], Ying-Ting Lee[1], Yi-An Chen[3], Yao-Chi Chen[4], Carmay Lim[4], Tzu-Han Hsu[2], Sung-Jan Lin[5,6,7], Hsin-Yung Yen[3], Kuo-Chiang Hsia[2,*], Su-Yi Tsai[1,7,8,*]

[1] Department of Life Science, National Taiwan University, Taipei, 10617, Taiwan
[2] Institute of Molecular Biology, Academia Sinica, Taipei 11529, Taiwan.
[3] Institute of Biological Chemistry, Academia Sinica, Taipei, Taiwan
[4] Institute of Biomedical Sciences, Academia Sinica, Taipei, Taiwan
[5] Department of Biomedical Engineering, College of Medicine and College of Engineering, National Taiwan University, Taipei, Taiwan
[6] Department of Dermatology, National Taiwan University Hospital and College of Medicine, Taipei, Taiwan
[7] Research Center for Developmental Biology and Regenerative Medicine, National Taiwan University, Taipei, 10617, Taiwan
[8] Genome and Systems Biology Degree Program, National Taiwan University, Taipei, 10617, Taiwan
[#] Equal contribution
[*]Correspondence should be addressed to K.-C. H. (e-mail: khsia@gate.sinica.edu.tw), and S.-Y. T. (e-mail: suyitsai@ntu.edu.tw)

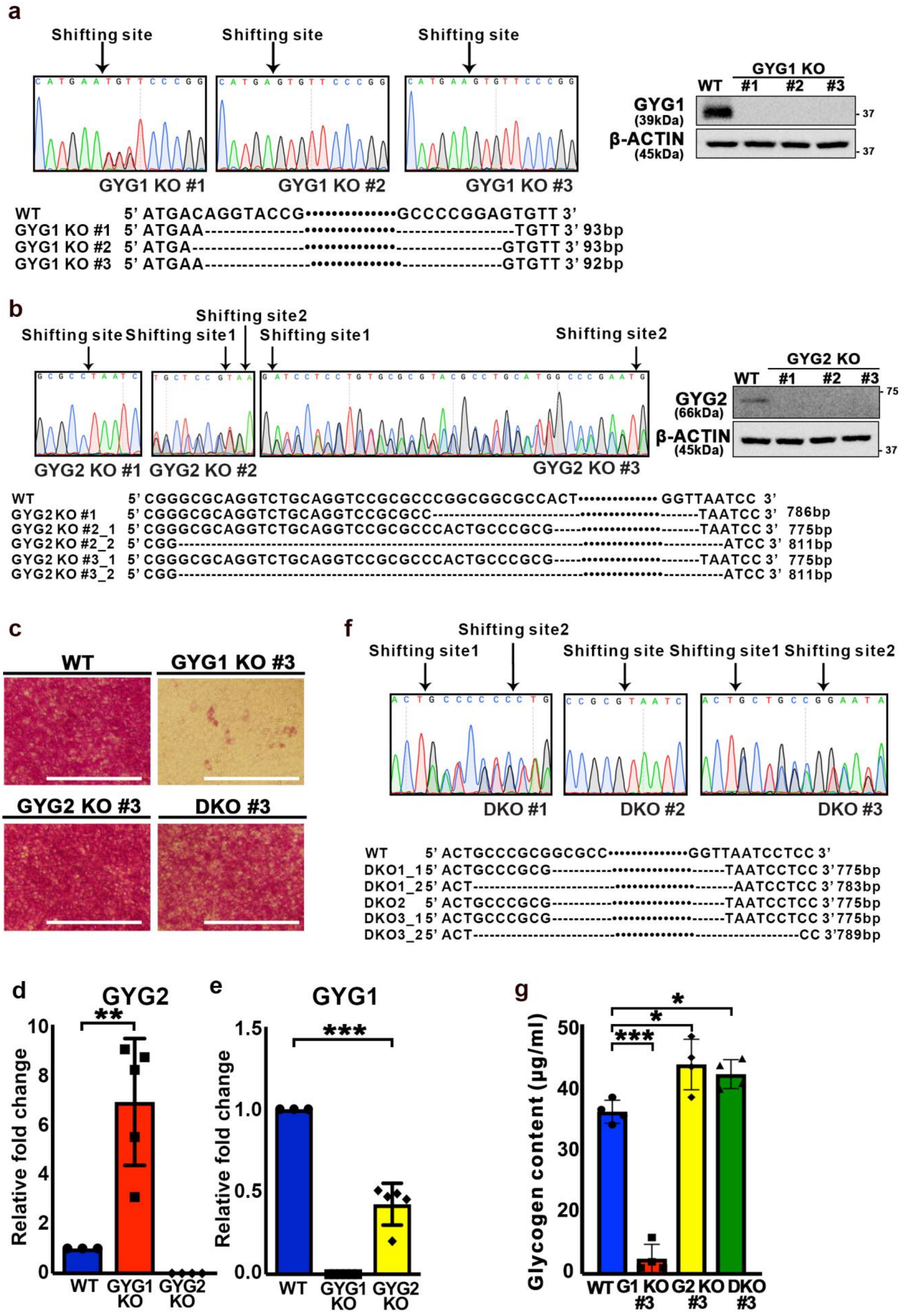
a
Shifting site
Shifting site
Shifting site
GYG1 KO #1
GYG1 KO #2
GYG1 KO #3
GYG1 KO
WT #1 #2 #3
GYG1 (39kDa)
β-ACTIN (45kDa)
37
37
WT 5' ATGACAGGTACCG••••••••••••••GCCCCGGAGTGTT 3'
GYG1 KO #1 5' ATGAA----------------••••••••••••••-------------------TGTT 3' 93bp
GYG1 KO #2 5' ATGA------------------••••••••••••••------------------GTGTT 3' 93bp
GYG1 KO #3 5' ATGAA-----------------••••••••••••••-----------------GTGTT 3' 92bp
b
Shifting site
Shifting site1
Shifting site2
Shifting site1
Shifting site2
GYG2 KO #1
GYG2 KO #2
GYG2 KO #3
GYG2 KO
WT #1 #2 #3
GYG2 (66kDa)
β-ACTIN (45kDa)
75
37
WT 5' CGGGCGCAGGTCTGCAGGTCCGCGCCCGGCGGCGCCACT•••••••••••••• GGTTAATCC 3'
GYG2 KO #1 5' CGGGCGCAGGTCTGCAGGTCCGCGCC---------------------------••••••••••••••-------TAATCC 3' 786bp
GYG2 KO #2_1 5' CGGGCGCAGGTCTGCAGGTCCGCGCCCACTGCCCGCG-----••••••••••••••-------TAATCC 3' 775bp
GYG2 KO #2_2 5' CGG--••••••••••••••------------ATCC 3' 811bp
GYG2 KO #3_1 5' CGGGCGCAGGTCTGCAGGTCCGCGCCCACTGCCCGCG-----••••••••••••••-------TAATCC 3' 775bp
GYG2 KO #3_2 5' CGG--••••••••••••••------------ATCC 3' 811bp
c
WT
GYG1 KO #3
GYG2 KO #3
DKO #3
f
Shifting site1
Shifting site2
Shifting site
Shifting site1
Shifting site2
DKO #1
DKO #2
DKO #3
WT 5' ACTGCCCGCGGCGCC••••••••••••••GGTTAATCCTCC 3'
DKO1_1 5' ACTGCCCGCG-----------••••••••••••••------TAATCCTCC 3' 775bp
DKO1_2 5' ACT---------------------------••••••••••••••-------AATCCTCC 3' 783bp
DKO2 5' ACTGCCCGCG-----------••••••••••••••------TAATCCTCC 3' 775bp
DKO3_1 5' ACTGCCCGCG-----------••••••••••••••------TAATCCTCC 3' 775bp
DKO3_2 5' ACT---------------------------••••••••••••••-------------------CC 3' 789bp
d
GYG2
Relative fold change
WT
GYG1 KO
GYG2 KO
e
GYG1
Relative fold change
WT
GYG1 KO
GYG2 KO
g
Glycogen content (μg/ml)
WT
G1 KO #3
G2 KO #3
DKO #3

Supplementary Figure 1. Glycogenin is undetectable in GYG1 KO, GYG2 KO and DKO hESCs

a-b, Left panels: Sequence electropherograms of the GYG1 (**a**) and GYG2 (**b**) knockout lines. Right panels: Western blot analysis of GYG1 and GYG2 expression in the GYG1 (**a**) and GYG2 (**b**) knockout hESCs. Wild type (WT) and three independent cell clones of each knockout line were examined. β-Actin served as the loading controls. CRISPR sgRNAs were designed to induce Cas9-mediated DNA double-strand breaks within selected sequences, leading to the deletion of the *GYG* genes. Three independent clones for each deletion mutant line are shown. **c,** Representative PAS staining images of WT, GYG1 KO #3, GYG2 #3, and DKO #3 hESCs. Scale bar: 200 μm. **d-e,** Quantification data of GYG2 (**d**) and GYG1 (**e**) expression in the GYG1 KO and GYG2 KO hESCs. Data are mean ± SD. One-way ANOVA (Tukey's multiple comparison test); Statistical significance is indicated: $^{**}p < 0.01$, $^{***}p < 0.001$. Five independent experiments were analyzed. **f**, Sequence electropherograms of the double knockout lines. **g,** Bar graph of glycogen content in indicated cells. Data represent mean ± SD. One-way ANOVA (Tukey's multiple comparison test); Statistical significance is indicated: $^{*}p < 0.05$, $^{***}p < 0.001$. Results represent data from four independent experiments.

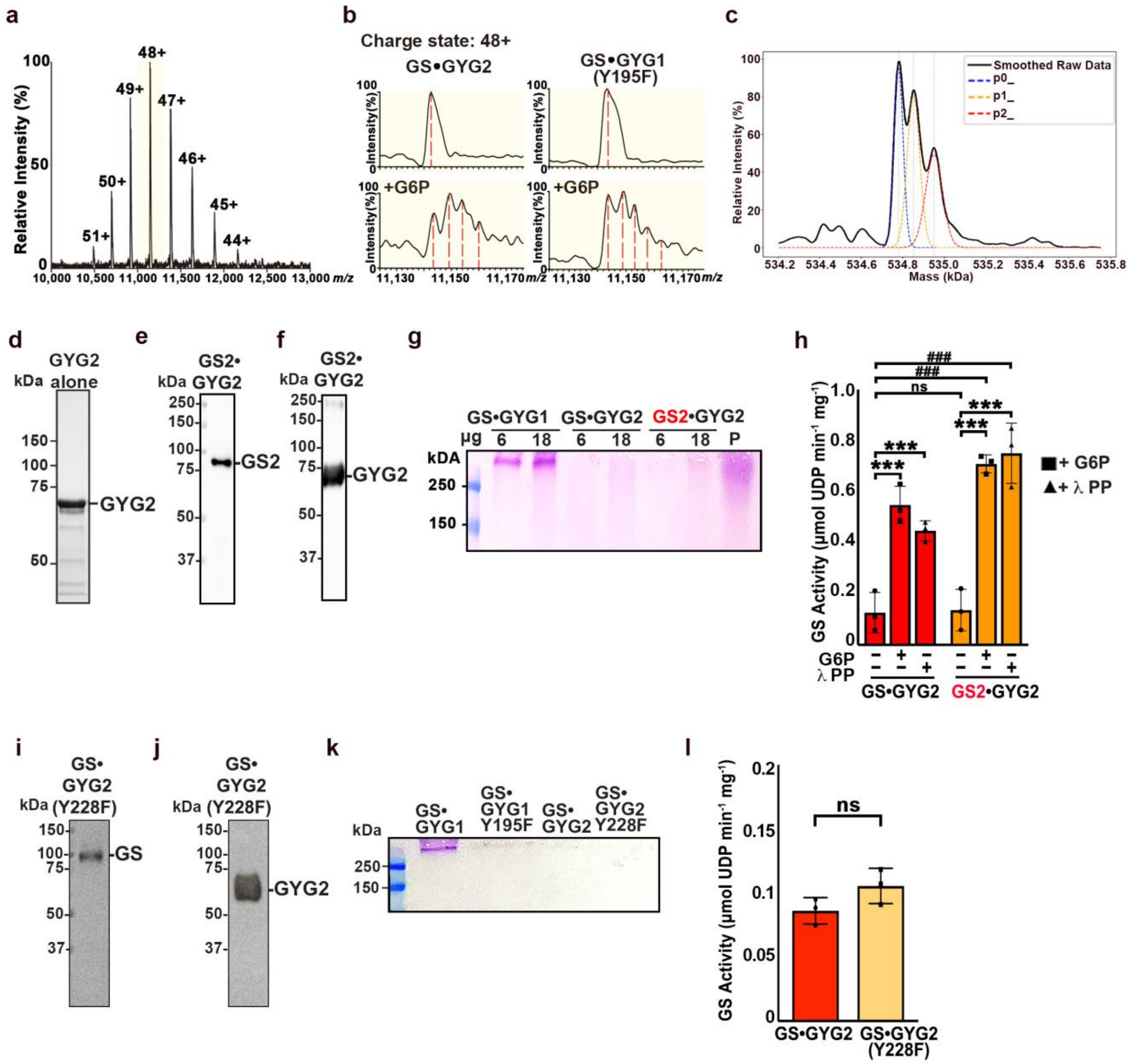

Supplementary Figure 2. Binding of G6P to the GS•GYG2 complex, as revealed by native mass spectrometry. **a,** A representative full MS spectrum of the GS•GYG2 complex, showing the consecutive charge distribution. **b,** Zoomed-in spectra of the GS•GYG2 complex in the 48^{+} charge state (yellow region in (**a**)) following co-incubation with G6P to resolve satellite signals corresponding to G6P binding (lower panel) by comparison to the spectra without G6P treatment (upper panel). **c,** The overlapping peaks in the deconvoluted mass spectrum were annotated by peak-shape fitting using Gaussian models. The centroid positions enabled measurement of a mass difference corresponding to the molecular weight of G6P. Observed molecular weights for complexes in the unbound and bound states are listed in Supplementary Table 4. **d,** Purified GYG2 alone was analyzed by SDS–PAGE and stained with Coomassie blue. **e-f,** Western blots of GS2 (**e**) and GYG2 (**f**) in GS2•GYG2 complex. **g,** Representative PAS staining of the GS•GYG1, GS•GYG2, and GS2•GYG2 complexes. At least three independent experiments were analyzed. **h**, GS activity of GS•GYG2, and GS2•GYG2 complexes under G6P or **λ**PP treatment. * represents statistical

comparisons relative to controls of each complex with or without G6P or **λ**PP. # represents statistical comparison of GS2•GYG2 with GS•GYG2. Data are mean ± SD. Two-way ANOVA (Tukey's multiple comparison test); Statistical significance is indicated: ***p < 0.001 and #p<0.001. Three independent experiments were analyzed. **i-j**, Western blot images of GS (**i**) and GYG2 (**j**) in GS•GYG2 (Y228F) complex. **k**, Representative PAS staining of the GS•GYG1, GS•GYG1(Y195F), GS•GYG2, and GS•GYG2 (Y228F) complexes. Three independent experiments were repeated in similar results. **l**, GS activity of the GS•GYG2 and GS•GYG2(Y228F) complexes. Data are mean ± SD. Three independent experiments were analyzed.

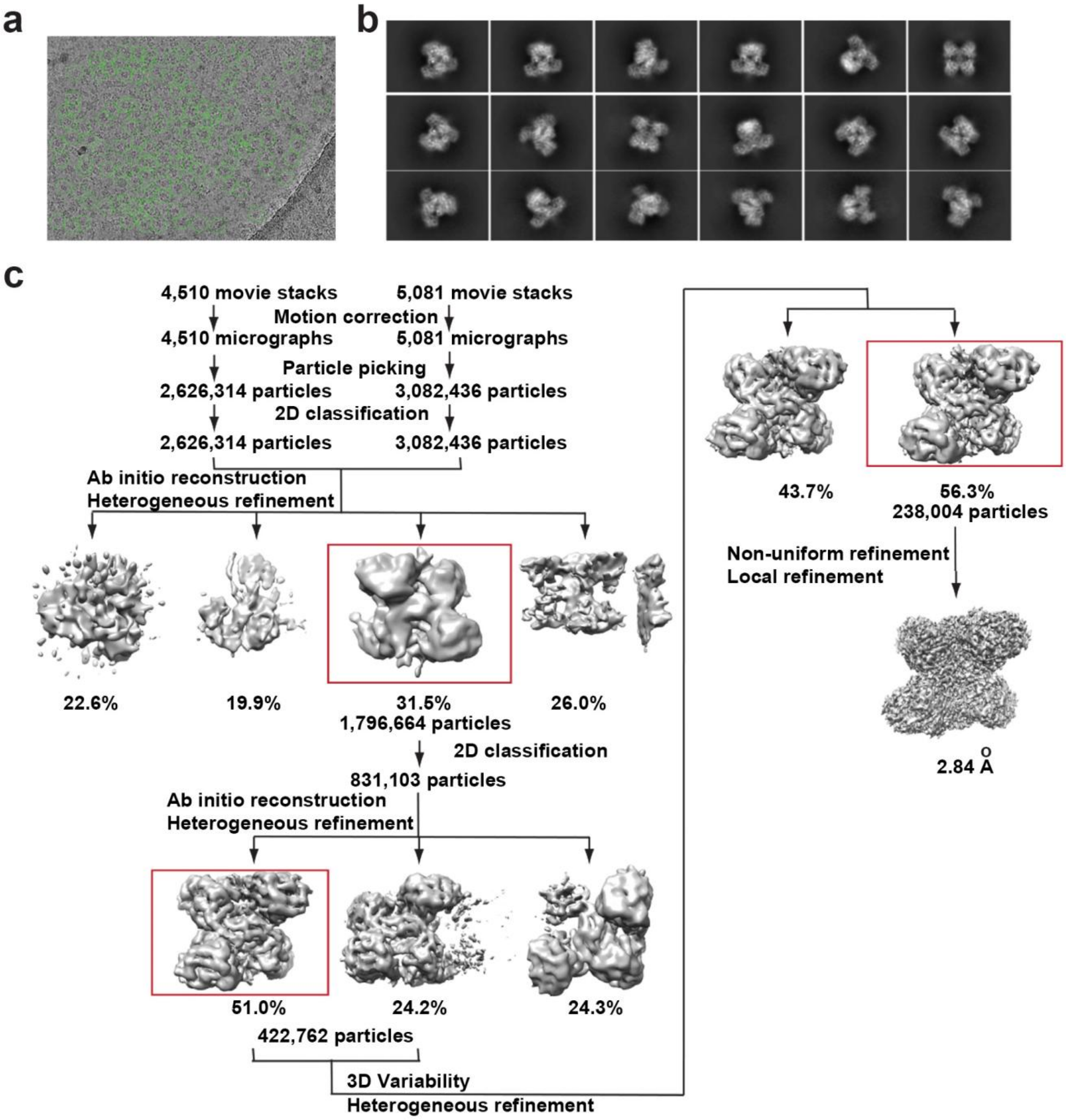

Supplementary Figure 3. Cryo-EM data processing of the GS•GYG2 complex. a, A representative cryo-electron micrograph of the GS•GYG2 complex particles. A field of the GS•GYG2 complex particles adsorbed onto a glow-discharged carbon grid and processed for imaging. Selected particles are highlighted by green circles. **b,** Representative reference-free 2D class-average images of the GS•GYG2 complex particles. **c,** Overview of the cryo-EM data processing pipeline for the GS•GYG2 complex. The micrographs were first processed using Relion and MotionCor2[1, 2]. Motion-corrected micrographs were then imported into cryoSPARC[3] for further single-particle reconstruction. 2D classification, *ab initio* model generation, 3D refinement (EMD-39700), and focus refinement were conducted using cryoSPARC.

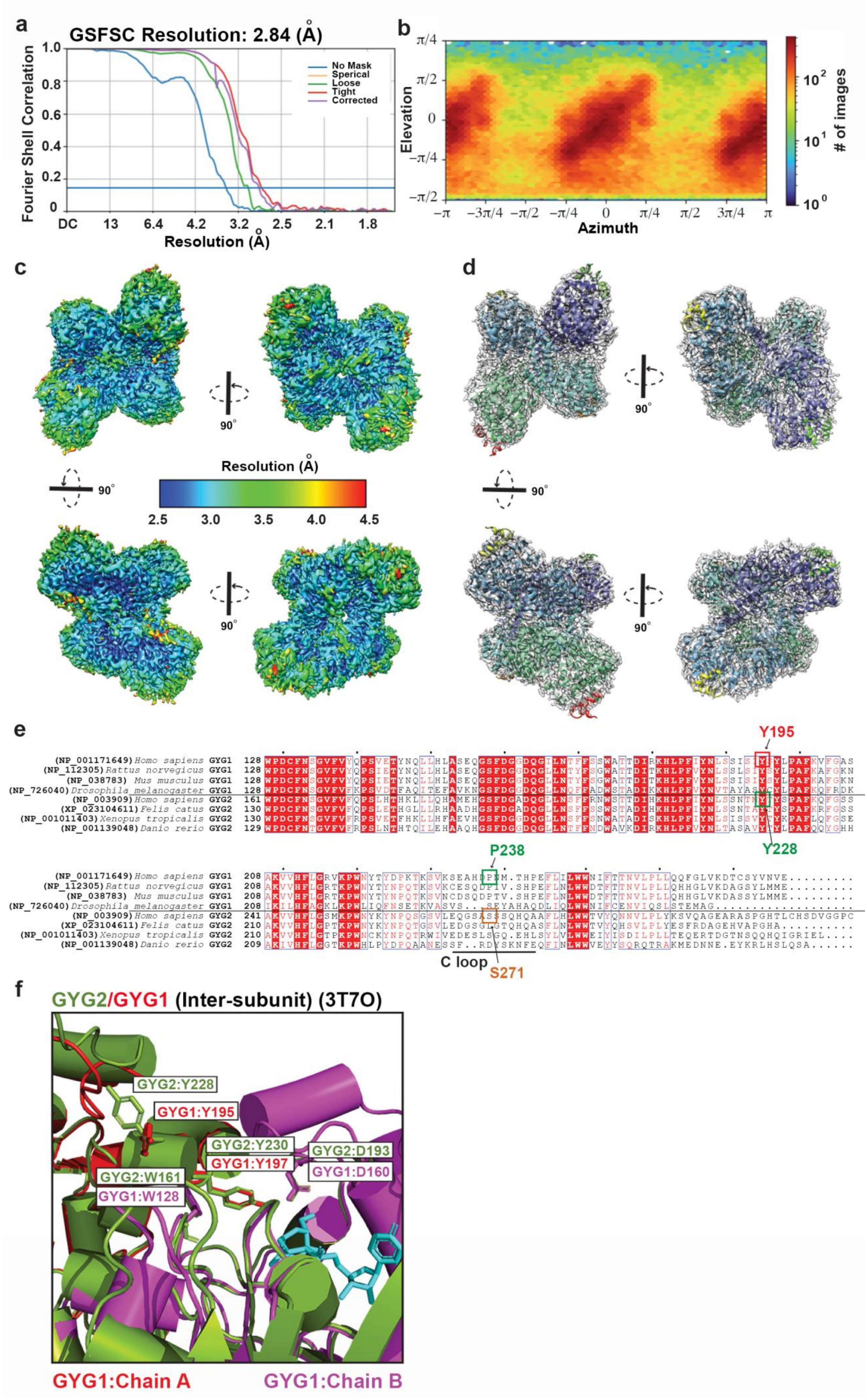
a
GSFSC Resolution: 2.84 (Å)
Fourier Shell Correlation
Resolution (Å)
No Mask
Sperical
Loose
Tight
Corrected
b
Elevation
Azimuth
# of images
c
Resolution (Å)
2.5 3.0 3.5 4.0 4.5
90°
d
e
Y195
Y228
P238
S271
C loop
(NP_001171649) Homo sapiens GYG1
(NP_112305) Rattus norvegicus GYG1
(NP_038783) Mus musculus GYG1
(NP_726040) Drosophila melanogaster GYG1
(NP_003909) Homo sapiens GYG2
(XP_023104611) Felis catus GYG2
(NP_001011403) Xenopus tropicalis GYG2
(NP_001139048) Danio rerio GYG2
f
GYG2/GYG1 (Inter-subunit) (3T7O)
GYG2:Y228
GYG1:Y195
GYG2:Y230
GYG1:Y197
GYG2:D193
GYG1:D160
GYG2:W161
GYG1:W128
GYG1:Chain A
GYG1:Chain B

Supplementary Figure 4. Cryo-EM results of the GS•GYG2 complex particles. a, The final resolution obtained from processing 238,004 particles, reported according to gold-standard Fourier Shell Correlation (FSC) with a cut-off of 0.143. **b,** The angular plot of the orientations calculated in cryoSPARC and assigned to the 238,004 particles. Heatmap showing the number of particles for each viewing angle. **c,** The cryo-EM map of the GS•GYG2 complex, colored based on local resolution. Four rotated views of the map are shown (EMD-39700). **d,** The cryo-EM structure of GS (PDB code: 7ZBN) and GYG2 (AlphaFold-predicted) were docked into the corresponding electron microscopy density map colored in transparent gray. Four rotated views are shown. **e,** Protein sequence alignment of the GYG1 and GYG2 N-terminal catalytic Rossmann fold domain across different species. The autoglycosylation sites Y195 in GYG1 and Y228 in GYG2 are indicated. Residues that are fully conserved or have similar properties are highlighted in red and boxed. The C loop residue P238 in GYG1 and its corresponding residue S271 in GYG2 are also marked. f, Superimposition of the AlphaFold3-predicted catalytic domain of human GYG2 (green) with the crystal structure of the GYG1 catalytic domain. Residues in GYG2 that mediate homodimeric interactions with an adjacent molecule are highlighted, along with their corresponding residues in GYG1.

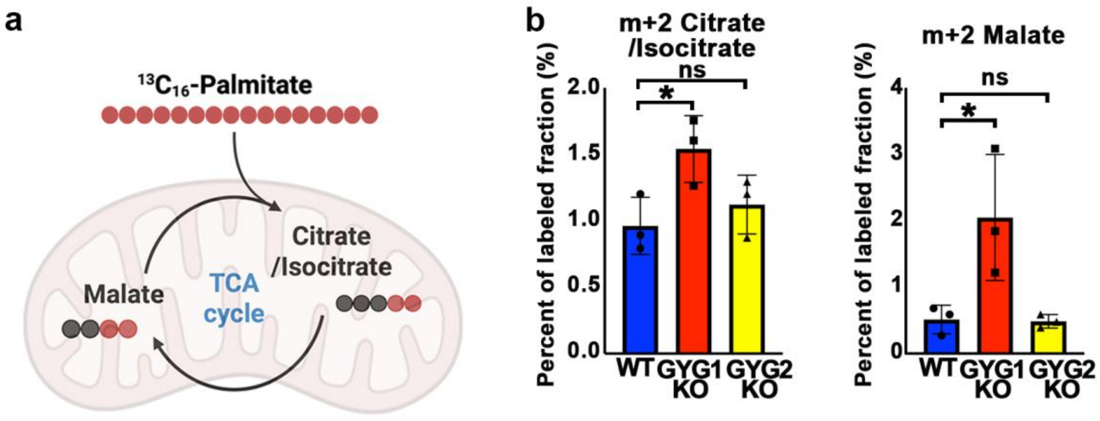

Supplementary Figure 5. Tracing of $^{13}C_{16}$-palmitate shows the metabolic shift in GYG1 KO cells. a, Graph of $^{13}C_{16}$-palmitate metabolic flux in the TCA cycle. The red circles represent labeled carbon. **b,** The percentage of labeled m+2 citrate/isocitrate and malate in WT, GYG1 KO, and GYG2 KO hESCs following 50 μM $^{13}C_{16}$-palmitate treatment for 16 hours. Three independent experiments were analyzed. Data are mean ± SD. Differences were assessed statistically using one-way ANOVA (Tukey's multiple comparison test); Statistical significance is indicated: * $p < 0.05$. Panel a was created with BioRender.com.

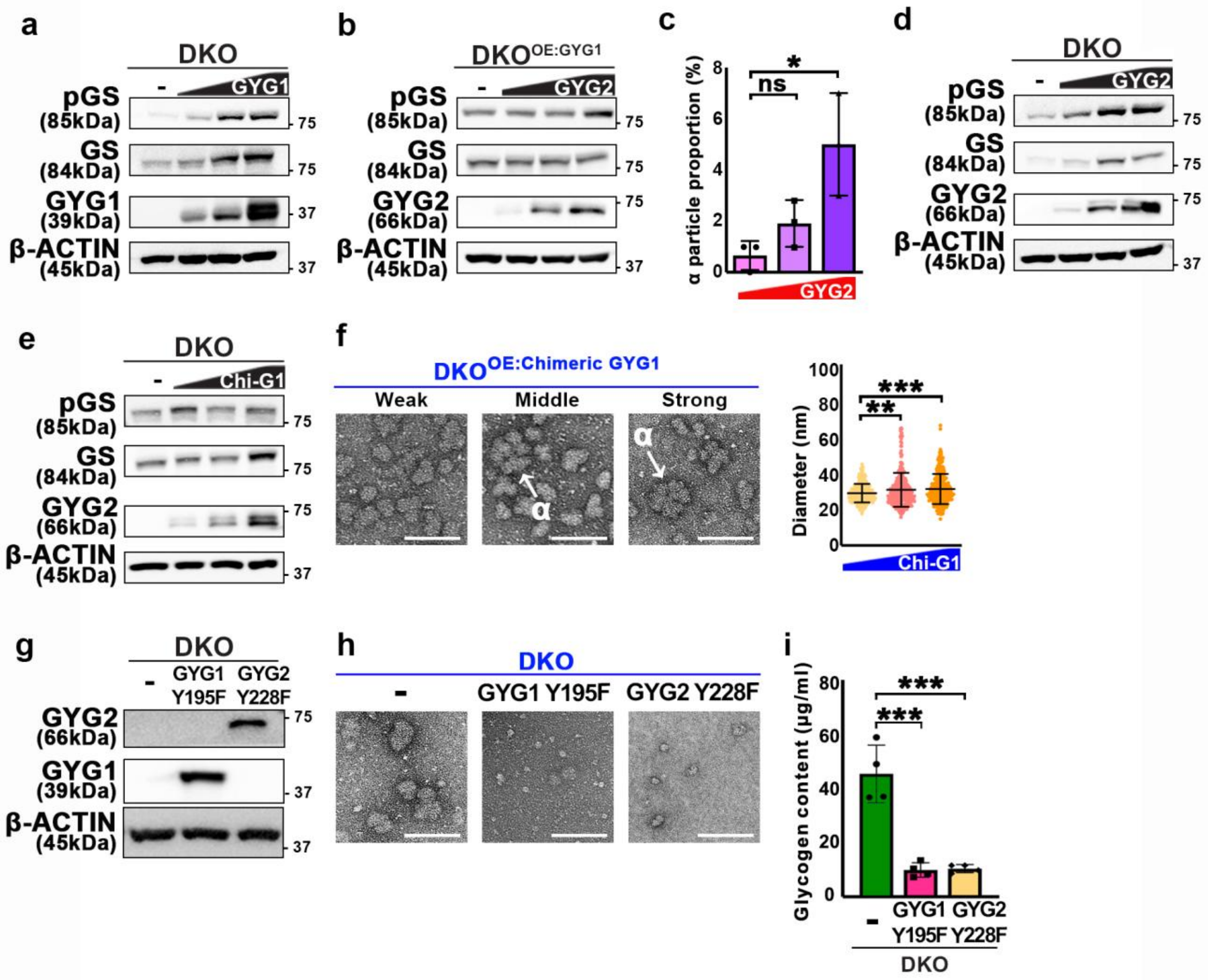

Supplementary Figure 6. Ectopic expression of GYG1 and GYG2 in DKO and DKO$^{OE:GYG1}$ hESCs. **a-e,** Western blot analysis of pGS, GS, GYG1 and GYG2 in the DKO$^{OE:GYG1}$ **(a)**, DKO$^{OE:GYG1\&2}$ **(b)**, DKO$^{OE:GYG2}$ **(d)**, and DKO$^{OE:chimeric\ GYG1}$ **(e)** lines. **c**, The proportion of α particles (>40 nm) in DKO$^{OE:GYG1\&2}$ lines. Data are mean ± SD. One-way ANOVA (Tukey's multiple comparison test); Statistical significance is indicated: *$p < 0.05$. At least 100 glycogen particles were analyzed in three independent experiments. **f,** Representative TEM images of glycogen particles isolated from DKO$^{OE:chimeric\ GYG1}$ hESCs subjected to three different concentrations (weak, middle, and strong) of chimeric GYG1. Quantification analysis of particle sizes is presented in the right panel and scale bars represent 100 nm. Data are mean ± SD. One-way ANOVA (Tukey's multiple comparison test); Statistical significance: **$p<0.01$ and ***$p < 0.001$ (n=at least 300 glycogen particles in total for each cell line). **g,** Western blot images of GYG1 and GYG2 expression in the DKO$^{OE:GYG1\ Y195F}$ and DKO$^{OE:GYG2\ Y228F}$ hESCs. **h,** Representative TEM images of glycogen particles isolated from DKO$^{OE:GYG1\ Y195F}$ and DKO$^{OE:GYG2\ Y228F}$ hESCs. Scale bar: 100 nm. **i,** Bar graph of glycogen content in the indicated cells. Data are representative of at least three independent experiments. Data are mean ± SD. Differences were assessed statistically using one-way ANOVA(Tukey's multiple comparison test); Statistical significance:***$p < 0.001$**.**

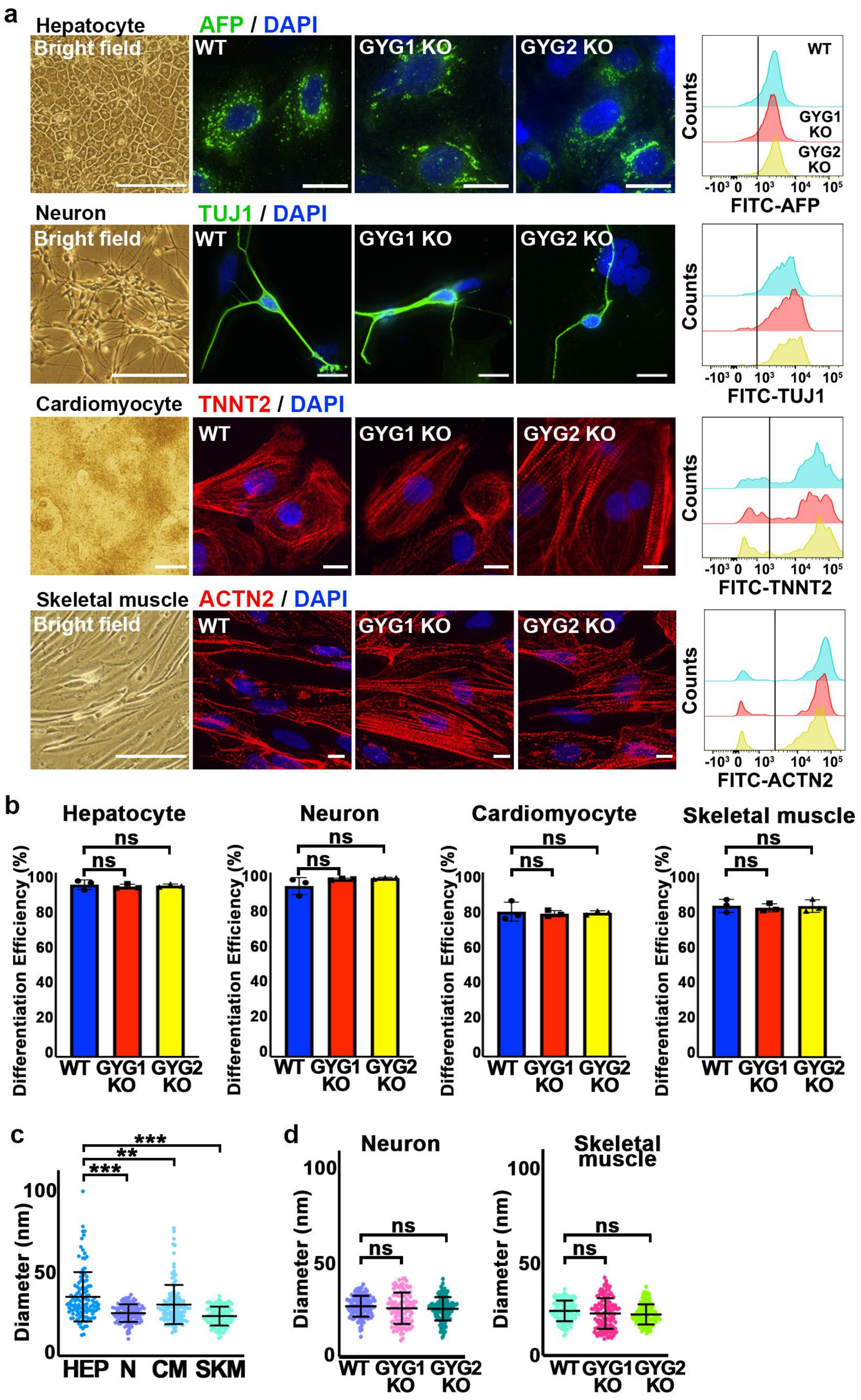
a
Hepatocyte
Bright field
AFP / DAPI
WT
GYG1 KO
GYG2 KO
Counts
GYG1 KO
GYG2 KO
FITC-AFP
Neuron
TUJ1 / DAPI
FITC-TUJ1
Cardiomyocyte
TNNT2 / DAPI
FITC-TNNT2
Skeletal muscle
ACTN2 / DAPI
FITC-ACTN2
b
Hepatocyte
Neuron
Cardiomyocyte
Skeletal muscle
ns
Differentiation Efficiency (%)
WT
GYG1 KO
GYG2 KO
c
Diameter (nm)
HEP
N
CM
SKM
***
**
d
Neuron
Skeletal muscle

Supplementary Figure 7. Glycogen particles and glycogen content in different cell lineages.

a, Left panel: Representative bright-field images of hESC-derived hepatocytes, neuronal cells, cardiomyocytes, and skeletal muscle. Scale bar: 200 μm. Middle panel: Immunofluorescence staining of the hepatocyte marker AFP, the neuronal marker TUJ1, cardiomyocyte marker TNNT2 and the skeletal muscle marker ACTN2. Scale bar: 20 μm. Right panel: Representative histograms of flow cytometry using different anti-FITC antibodies conjugated with indicated markers. Data represent one representative group from three independent experiments with similar results. **b,** Plots of the differentiation efficiency of hepatocytes, neurons, cardiomyocytes, and skeletal muscle, as determined by flow cytometry. Data are representative of three independent experiments. Data are mean ± SD. Differences were assessed statistically using one-way ANOVA (Tukey's multiple comparison test). **c,** Plot of wildtype glycogen particle diameter in the indicated cell lineages. Data are mean ± SD. Differences were assessed statistically using one-way ANOVA (Tukey's multiple comparison test); ** $p < 0.01$, *** $p < 0.001$. N=at least 100 glycogen particles in each cell lineages. **d,** Plot of glycogen particle diameter in the indicated hESC-derived neuron cells and skeletal muscle. Data are mean ± SD. Differences were assessed statistically using one-way ANOVA (Tukey's multiple comparison test); ** $p < 0.01$, *** $p < 0.001$. N=at least 100 glycogen particles in each cell lines.

Supplementary Table 1 Cryo-EM data collection, refinement and validation statistics

	**GS•GYG2 (EMD-39700) (8Z0A)**	**GS•GYG2-Tilt**
**Data collection**		
EM equipment	Titan Krios	Titan Krios
Voltage (kV)	300	300
Cs (mm)	2.7	2.7
Magnification (nominal)	105,000	105,000
Detector	K3	K3
Pixel size (Å)	0.83	0.83
Electron exposure (e^{-}/Å^{2})	~ 50.23	~ 49.69
Exposure time (s)	1.62	1.62
Frames (no.)	50	60
Defocus range (μm)	-1.2 ~ -1.8	-1.2 ~ -1.8
Tilt angle(˚)	-	20˚
Micrographs stacks (no.)	4,510	5,081
**Reconstruction**		
Software	cryoSPARC	
Final particle images (no.)	238,004	
Symmetry imposed	D2	
Map final resolution (Å) †	2.84	
Map sharpening B-factor (Å2)	-130.2	
**Atomic modeling**		
Software	Coot & Phenix	
Number of protein residues	2544	
Number of ligands	0	
Number of atoms	20668	
Mask CC *	0.82	
Volume CC *	0.78	
RMSD bond lengths (Å)	0.004	
RMSD bond angles (°)	0.533	
Clash score *	7.75	
Ramachandran favored (%) *	97.30	
Ramachandran allowed (%) *	2.70	
Ramachandran outliers (%) *	0.00	
Rotamer outliers (%) *	0.00	
C_{β} deviations *	0.00	
MolProbity score *	1.56	

†According to FSC=0.143

* According to the criterion of Chen *et al.*, 2010 [4].

Supplementary Table 2. Primer lists.

Name of Primer	Sequence (5' to 3')
GYG1 sgRNA #1_F	GCTGCGCGGCGGTACCTGTCA
GYG1 sgRNA #1_R	TGACAGGTACCGCCGCGCAGC
GYG1 sgRNA #2_F	GTCCTGCCGGGAACACTCCG
GYG1 sgRNA #2_R	CGGAGTGTTCCCGGCAGGAC
GYG1 genotype_F	GGCTTCTCTGAGTCACCAACCTG
GYG1 genotype_R	GGACGCCTCGCAACAAGTGAAG
GYG2 sgRNA #1_F	GACATGGTCAGTGGCGCCGC
GYG2 sgRNA #1_R	GCGGCGCCACTGACCATGTC
GYG2 sgRNA #2_F	GTGCCTGCAGGAGGATTAACC
GYG2 sgRNA #2_R	GGTTAATCCTCCTGCAGGCAC
GYG2 genotype_F	CAGGTGACATGGAGTGGGTAG
GYG2 genotype_R	GATCCAGTGTGGTCTGGTACAAG
GYG2 OE_NheI_F	GCTAGCATGTCGGAGACAGAGTTTCACCATGG
GYG2 OE_EcoRI_R	GAATTCTTACTGCAGGAACCGGTCCAGCTTC
GYG2 GSD sgRNA#1 F	AGAGACGGCCAGGTCGACCT
GYG2 GSD sgRNA#1 R	AGGTCGACCTGGCCGTCTCT
GYG2 GSD sgRNA#2 F	GGACGCGTTTGCTCGCATCC
GYG2 GSD sgRNA#2 R	GGATGCGAGCAAACGCGTCC
GYG2 GSD genotype_F	GAGCCCCATGGTAGGTGGATG
GYG2 GSD genotype_R	CCATTTAGCACACCGAGGGGC
GYG1 OE Rossmann_NheI_F	GCTAGCATGACAGATCAGGCCTTTGTGAC
GYG1 OE Rossmann_KpnI_R	GGTACCTTGAAGCAGAGGTAAAACGTTGGTG
GYG1 OE Link_KpnI_F	GGTACCCAATTTGGCCTTGTCAAAGACACCTGC

GYG1 OE Link_BamHI_R	GGATCCCTTTCTACAGAAGCCACAAGAGAAAGCC
GYG1 OE GSD_BamHI_F	GGATCCGAAGATGTCTCAGGAGCCATATCAC
GYG1 OE GSD_NotI_R	GCGGCCGCCTGGAGGTAAGTGTCAAGTTTCC
GYG2 OE Rossmann_KpnI_R	GGTACCCTGGTAGACCGTCCACCAGAGATG
GYG2 OE Link_KpnI_F	GGTACCAACAACGTGCTGCCCCTTTATAAAAGCG
GYG2 OE Link_BamHI_R	GGATCCCTCCAGTGCATCCTGCAGACTG
GYG2 OE GSD_BamHI_F	GGATCCGTCGACCTGGCCGTCTCTGTTTC
GYS2_NheI_F	GCTAGCATGCTTCGAGGCCGATCCCTC
GYS2_NdeI_R	CATATGTTCAGCTTCTATTGCTGTTATTTCAGAAACCG
GYS2_NdeI_F	CATATGCTGAAGAGAAAGCCTGATGTAGTTAC
GYS2_NotI_R	GCGGCCGCGTTCTTATATTCACCATGCAGCTTTTTC
FLAG_EcoRI_R	GAATTCCTATTTATCGTCATCATCTTTGTAGTCCTTG
pLAS3W sequence	GTTCGGCTTCTGGCGTGTG
pX330 sequence	GGACTATCATATGCTTACCG

Supplementary Table 3. Primary antibodies

Target antigen	Vendor or Source	Catalog #	Species	Working concentration	Application
Glycogenin-1 (GYG1)	SantaCruz	Sc271109	Ms	1:1000	WB
Glycogenin-2 (GYG2)	SantaCruz	Sc134346	Ms	1:500	WB
Glycogen synthase	Cell Signaling	3893	Rb	1:1000	WB
Phospho-glycogen synthase (Ser641)	Cell Signaling	3891	Rb	1:1000	WB
Glycogen synthase 2	SantaCruz	Sc390391	Ms	1:1000	WB
FLAG	GeneTex	GTX115043	Rb	1:1000	WB
Alpha-tubulin	GeneTex	GTX628802	Ms	1:10000	WB
Beta-actin	Taiclone	Tcba13636	Rb	1:10000	WB
AFP	SantaCruz	Sc8399	Ms	1:200	IF
TUJ1	GeneTex	GTX631836	Ms	1:1000	IF
TNNT2	abcam	ab45932	Rb	1:800	IF
ACTN2	Invitrogen	710947	Ms	1:500	IF
AFP-FITC	SantaCruz	Sc8399-FITC	Ms	1:100	Flow
Beta-tubulin-FITC	SantaCruz	Sc5274-FITC	Ms	1:100	Flow
TNNT2-FITC	SantaCruz	Sc20025-FITC	Ms	1:100	Flow
ACTN2-FITC	SantaCruz	Sc17829-FITC	Ms	1:100	Flow

Ms, mouse; Rb, rabbit; WB, Western blotting; IF, Immunofluorescence.

Supplementary Table 4. The deconvoluted masses derived from the m/z spectrum

Peak	mean	ΔMass (Da) between peaks	SEM	Binding stoichiometry
**GS•GYG2 complex**				
Apo	534837.8±10.5			0
1st	535095.1±27.7	257.3	6.07	1
2nd	535370.2±40.9	275.2	15.9	2
**GS•GYG1 (Y195F) complex**				
Apo	534845.9±19.7			0
1st	535073.8±33.9	227.9	23.3	1
2nd	535265.5±30.8	191.7	18.5	2

1. Zheng X*, et al.* Molecular basis for CPAP-tubulin interaction in controlling centriolar and ciliary length. *Nat Commun* **7**, 11874 (2016).

2. Zivanov J*, et al.* New tools for automated high-resolution cryo-EM structure determination in RELION-3. *Elife* **7**, (2018).

3. Punjani A, Rubinstein JL, Fleet DJ, Brubaker MA. cryoSPARC: algorithms for rapid unsupervised cryo-EM structure determination. *Nat Methods* **14**, 290-296 (2017).

4. Chen VB*, et al.* MolProbity: all-atom structure validation for macromolecular crystallography. *Acta Crystallogr D Biol Crystallogr* **66**, 12-21 (2010).
