# Supplementary material for: Human glycogenins maintain glucose homeostasis by regulating glycogen metabolism
Source: Nat Commun. 2025 Jul 16;16:6556. doi: 10.1038/s41467-025-61862-3 (PMC12267440; doi:10.1038/s41467-025-61862-3)
Supplement: Supplementary file 2 — Reporting Summary [file 41467_2025_61862_MOESM2_ESM.pdf]

nature portfolio

Corresponding author(s): Su-Yi Tsai

Last updated by author(s): Jun 6, 2025

Reporting Summary

Nature Portfolio wishes to improve the reproducibility of the work that we publish. This form provides structure for consistency and transparency in reporting. For further information on Nature Portfolio policies, see our Editorial Policies and the Editorial Policy Checklist.

Statistics

For all statistical analyses, confirm that the following items are present in the figure legend, table legend, main text, or Methods section.

n/a	Confirmed	
☐	☒	The exact sample size (*n*) for each experimental group/condition, given as a discrete number and unit of measurement
☐	☒	A statement on whether measurements were taken from distinct samples or whether the same sample was measured repeatedly
☐	☒	The statistical test(s) used AND whether they are one- or two-sided<br>*Only common tests should be described solely by name; describe more complex techniques in the Methods section.*
☒	☐	A description of all covariates tested
☐	☒	A description of any assumptions or corrections, such as tests of normality and adjustment for multiple comparisons
☐	☒	A full description of the statistical parameters including central tendency (e.g. means) or other basic estimates (e.g. regression coefficient) AND variation (e.g. standard deviation) or associated estimates of uncertainty (e.g. confidence intervals)
☐	☒	For null hypothesis testing, the test statistic (e.g. *F*, *t*, *r*) with confidence intervals, effect sizes, degrees of freedom and *P* value noted<br>*Give P values as exact values whenever suitable.*
☒	☐	For Bayesian analysis, information on the choice of priors and Markov chain Monte Carlo settings
☒	☐	For hierarchical and complex designs, identification of the appropriate level for tests and full reporting of outcomes
☒	☐	Estimates of effect sizes (e.g. Cohen's *d*, Pearson's *r*), indicating how they were calculated

Our web collection on statistics for biologists contains articles on many of the points above.

Software and code

Policy information about availability of computer code

Data collection	For glycogen particle data collection: Talos transmission electron microscope (Thermo Scientific) equipped with a Ceta CMOS camera.<br>For cryo-EM data collection: Talos Arctica transmission electron microscope equipped with a Falcon III detector (Thermo Fisher Scientific) operated in linear mode. For high resolution data collection: 300 keV Titan Krios transmission electron microscope (Thermo Fisher Scientific) hosting a K3 detector (with GIF Bio-Quantum Energy Filters, Gatan) operating in super-resolution mode and using EPU-3.3.1.5184REL software (Thermo Fisher Scientific).
Data analysis	For statistical analyses: Prism 9.<br>For glycogen particle acquisition: TEM Imaging & Analysis, TIA (version 4.18)<br>For glycogen particle quantification analysis: ImageJ (version 1.53k)<br>For cryo-EM single particle analysis: Motion-correction and dose-weighting: MotionCor2 ; Single-particle reconstruction: cryoSPARC version 4.1<br>For atomic model building : COOT version 9.8

For manuscripts utilizing custom algorithms or software that are central to the research but not yet described in published literature, software must be made available to editors and reviewers. We strongly encourage code deposition in a community repository (e.g. GitHub). See the Nature Portfolio guidelines for submitting code & software for further information.

Data

Policy information about availability of data

All manuscripts must include a data availability statement. This statement should provide the following information, where applicable:

- Accession codes, unique identifiers, or web links for publicly available datasets
- A description of any restrictions on data availability
- For clinical datasets or third party data, please ensure that the statement adheres to our policy

The data supporting the findings from this study are available within the manuscript and its supplementary information. The source data underlying panels in Figure. 1–7 and Supplementary Figure 1, 2, 5, 6, and 7 are provided in a source data file. Protein structural coordinates and maps have been deposited in the PDB and the Electron Microscopy Data Bank (EMD) with the accession codes PDB 8Z0A, EMD-39700. Native mass spectrometry for G6P binding have been have been deposited at Zenodo (https://doi.org/10.5281/zenodo.15742562). The metabolomics data have been deposited to MetaboLights repository with the study identifier MTBLS12586. All other data are available from the corresponding author upon request. Source Data are provided with this paper.

Research involving human participants, their data, or biological material

Policy information about studies with human participants or human data. See also policy information about sex, gender (identity/presentation), and sexual orientation and race, ethnicity and racism.

Reporting on sex and gender	N/A
Reporting on race, ethnicity, or other socially relevant groupings	N/A
Population characteristics	N/A
Recruitment	N/A
Ethics oversight	N/A

Note that full information on the approval of the study protocol must also be provided in the manuscript.

Field-specific reporting

Please select the one below that is the best fit for your research. If you are not sure, read the appropriate sections before making your selection.

☒ Life sciences ☐ Behavioural & social sciences ☐ Ecological, evolutionary & environmental sciences

For a reference copy of the document with all sections, see nature.com/documents/nr-reporting-summary-flat.pdf

Life sciences study design

All studies must disclose on these points even when the disclosure is negative.

Sample size	No method was used to calculate the sample size.
Data exclusions	No data were excluded.
Replication	At least three independent replicates were performed for each quantification analysis. For glycogen particles quantification, at least thre hundred independent particles are involved.
Randomization	No randomization was involved.
Blinding	No blinding was involved.

Reporting for specific materials, systems and methods

We require information from authors about some types of materials, experimental systems and methods used in many studies. Here, indicate whether each material, system or method listed is relevant to your study. If you are not sure if a list item applies to your research, read the appropriate section before selecting a response.

Materials & experimental systems

n/a	Involved in the study
☐	☒ Antibodies
☐	☒ Eukaryotic cell lines
☒	☐ Palaeontology and archaeology
☒	☐ Animals and other organisms
☒	☐ Clinical data
☒	☐ Dual use research of concern
☒	☐ Plants

Methods

n/a	Involved in the study
☒	☐ ChIP-seq
☒	☐ Flow cytometry
☒	☐ MRI-based neuroimaging

Antibodies

Antibodies used

Antibody details are described in the materials and method section. Following are the details of the antibodies:
anti-AFP(Santa Cruz, sc-8399, 1:200, IF)
anti-TUJ1 (GeneTex, GTX631836,1:1000, IF)
anti-TNNT2 (abcam, ab45932, 1:800, IF)
anti-ACTN2 (Invitrogen, 710947,1:500, IF)
anti-GYG1 (Santa Cruz, sc-271109, 1:1000, Western Blot)
anti-GYG2 (Santa Cruz, sc-134346, 1:1000, Western Blot)
anti-glycogen synthase (Cell Signaling, 3893, 1:1000, Western Blot)
anti-glycogen synthase 2 (Santa Cruz, sc-390391, 1:1000, Western Blot)
anti-phospho-glycogen synthase (Ser641) (Cell Signaling, 3891, 1:1000, Western Blot)
anti-beta-Actin (Taiclone, tcba13636, 1:10000, Western Blot)
anti-alpha-Tubulin (GeneTex, GTX628802,1:1000, Western Blot)
anti-Vinculin (Sigma-Aldrich, V9131, 1:1000, Western Blot)
anti-flag (GeneTex, GTX115043, 1:1000, Western Blot)-
anti-AFP-FITC (Santa Cruz, sc8399-FITC, 1:100, Flow cytometry)
anti-beta tubulin-FITC (Santa Cruz, sc5274-FITC, 1:100, Flow cytometry)
anti-TNNT2-FITC (Santa Cruz, sc-20025FITC, 1:100, Flow cytometry)
anti-ACTN2-FITC (Santa Cruz, sc17829-FITC, 1:100, Flow cytometry)

Validation

All antibodies were commercially validates as below:
All primary antibodies used in this study were commercially obtained and validated by the manufacturers for Western blotting or immunostaining in human or mammalian cells. Validation information is available on the suppliers' websites and/or previously published literature. The specific details are as follows:
anti-AFP (Santa Cruz, sc-8399) validated by immunofluorescence in human cells and referenced in multiple studies. https://www.scbt.com/p/afp-antibody-c3?srsltid=AfmBOorBQBo6bRAMr4zyXsADfnmuJOLMpckl1ZV16TXkq976IY6ySsWC

anti-TUJ1 (GeneTex, GTX631836) validated by immunofluorescence in mammalian tissues and referenced in multiple studies. https://www.genetex.com/Product/Detail/beta-Tubulin-3-Tuj1-antibody GT11710/GTX631836?srsltid=AfmBOopoSEKu7qX6ixzIqoRjAVZp_kjaRXIZz1NQqPVqQ94bZoAD7hVq

anti-TNNT2 (abcam, ab45932) validated by abcam for WB/IHC in human cell lysate and tissues and cited by over 100 studies. https://www.abcam.com/en-us/products/primary-antibodies/cardiac-troponin-t-antibody-ab45932?srsltid=AfmBOorAKfeK77IBHKM0i2-Cj0UeCiiYgMEx6fQ2BT8qCjA60lxOdgrL

anti-ACTN2 (Invitrogen, 710947) validated for IHC/IF in human cells. https://www.thermofisher.com/antibody/product/alpha-Actinin-2-Antibody-Recombinant-Superclonal/710947

anti-GYG1 (Santa Cruz, sc-271109) validated for WB in several human cell lysates. https://www.scbt.com/p/glycogenin-1-antibody-e-11?srsltid=AfmBOoq23aQf33WGkuSN3ZHN4FWQBwOhfUb_57Wa_Vr7YGSzHrcufA0B

anti-GYG2 (Santa Cruz, sc-134346) validated for WB in several human cell lysates. https://www.scbt.com/p/glycogenin-2-antibody-3l10?srsltid=AfmBOorTgZTx7Cgksd7VwCfDcCwFYVRgBAYassUMBIXRJo3uwm5blYEt

anti-glycogen synthase (Cell Signaling, 3893) Validated for Western blotting in human and rodent tissues. Cited in multiple peer-reviewed publications. https://www.cellsignal.com/products/primary-antibodies/glycogen-synthase-1-gys1-antibody/3893?srsltid=AfmBOophC-czU3Vwe016cmyaFlQ5Maxw4I7BWac_I_pz-2Gsd0mVaDWe

anti-glycogen synthase 2 (Santa Cruz, sc-390391) validated for WB in human tissues. https://www.scbt.com/p/glycogen-synthase-2-antibody-g-8?srsltid=AfmBOooDz4N1frhlgPZSZiASPktfRgAC-LpS8J4JPncPF0Woq_wbZJQf

anti-phospho-glycogen synthase (Ser641) (Cell Signaling, 3891) validated by CST for WB in human/mouse/rat cells and cited in >150 publications. https://www.cellsignal.com/products/primary-antibodies/phospho-glycogen-synthase-ser641-antibody/3891?srsltid=AfmBOoqRPPb41OZZlrkMhJ056tFogARGmyR1DCgv6xYI5uM80YCCGi_4

anti-beta-Actin (Taiclone, tcba13636) validated for WB in human cells and tissues. https://taiclone.com/product/214631/actin-monoclonal-antibody

anti-alpha-Tubulin (GeneTex, GTX628802) validated for WB in human cells and tissues. https://www.genetex.com/Product/Detail/

alpha-Tubulin-antibody-GT114/GTX628802?srsltid=AfmBOopeAffudg9aK-Gw2nbyXb4Y646QuOXebXIlN3T0SdW0QvLfg8pK

anti-Vinculin (Sigma-Aldrich, V9131) validated for WB in human cells and tissues and cited in several studies. https://www.sigmaaldrich.com/TW/zh/product/sigma/v9131?srsltid=AfmBOooVP3ViPR_Q0ACUP0edvcRvFCF76rcljf_Xg5LNSFYAHHB6sqjF
anti-flag (DDDDK tag) (GeneTex, GTX115043) validated for WB in human cells. https://www.genetex.com/Product/Detail/DDDDK-tag-antibody/GTX115043?srsltid=AfmBOooKeJpZ3IKPgS4mcJoRz7dtk0FKm_qpynhCkmNMsJQ577VYRxY8

anti-AFP-FITC (Santa Cruz, sc8399-FITC) can be found at manufacturer's website-https://www.scbt.com/p/afp-antibody-c3
anti-beta tubulin-FITC (Santa Cruz, sc5274-FITC) can be found at manufacturer's website-https://www.scbt.com/p/beta-tubulin-antibody-d-10

anti-TNNT2-FITC (Santa Cruz, sc-20025) can be found at manufacturer's website-https://www.scbt.com/p/troponin-t-c-antibody-ct3

anti-ACTN2-FITC (Santa Cruz, sc17829-FITC) can be found at manufacturer's website-https://www.scbt.com/p/alpha-actinin-antibody-h-2

Eukaryotic cell lines

Policy information about cell lines and Sex and Gender in Research

Cell line source(s)

Human H9 ESC lines were purchased from WiCell (WAe009-A).

Authentication

The H9 hESC line (WAe009-A) was obtained from WiCell and authenticated by the supplier using STR profiling.

Mycoplasma contamination

all cell lines tested negative for mycoplasma contamination

Commonly misidentified lines
(See ICLAC register)

N/A

Plants

Seed stocks

No plant was used in this study.

Novel plant genotypes

No plant was used in this study.

Authentication

No plant was used in this study.
